# Systemic Treatment of Locally Advanced or Metastatic Non-Clear Cell Renal Cell Carcinoma

**DOI:** 10.3390/cancers17091527

**Published:** 2025-04-30

**Authors:** Joseph Vento, Tian Zhang, Payal Kapur, Hans Hammers, James Brugarolas, Qian Qin

**Affiliations:** 1 Division of Hematology and Oncology, Department of Internal Medicine, University of Texas Southwestern, Dallas, TX 75235, USA; 2 Department of Pathology, University of Texas Southwestern, Dallas, TX 75235, USA

**Keywords:** non-clear cell renal cell carcinoma, papillary renal cell carcinoma, chromophobe renal cell carcinoma, unclassified renal cell carcinoma/renal cell carcinoma not further classified, translocation renal cell carcinoma, collecting duct renal cell carcinoma, renal medullary carcinoma

## Abstract

Non-clear cell renal cell carcinoma (nccRCC) represents a heterogenous group of malignancies with varying degrees of clinical aggressiveness and response to different systemic therapies. As the characterization of subtypes of nccRCC continues to evolve, it is important to understand the evidence around systemic treatments used in advanced or metastatic stages of specific subtypes. Here, we review the literature on systemic therapies in nccRCC, with a focus on prospective trials that included patients with papillary renal cell carcinoma (RCC), chromophobe RCC, RCC not further classified/unclassified RCC, translocation RCC, collecting duct RCC, and renal medullary carcinoma. We also review emerging treatments for other molecularly defined subtypes of this disease.

## Simple Summary

Non-clear cell renal cell carcinoma represents a diverse group of rare kidney cancers. As an increased number of treatments emerge, it is essential to understand systemic options for these cancers. Here, we review systemic therapies in non-clear cell renal cell carcinoma, with special attention to recent prospective clinical trials and treatment options for specific disease subtypes.

## 1. Introduction

The most common subtype of renal cell carcinoma (RCC) in adults is clear cell renal cell carcinoma (ccRCC), which makes up 75–80% of cases [1]. All other RCCs are grouped under the umbrella term of non-clear cell renal cell carcinoma (nccRCC), which encompasses a heterogenous group of malignancies that vary in characteristics such as cell of origin, morphology, and molecular features. Common subtypes included in nccRCC are papillary RCC (10–15% of all RCCs), chromophobe RCC (5–10%), and RCC not further classified (NOS) (previously referred to as unclassified RCC) (5%), while less common subtypes include collecting duct carcinoma (2%) and SMARCB1-deficient renal medullary carcinoma (<1%). The most recent 2022 World Health Organization (WHO) classification of kidney cancers included for the first time a category of molecularly defined subtypes of RCCs, which includes TFE3-rearranged RCC and TFEB-altered RCC (translocation RCC), fumarate hydratase (FH)-deficient RCC, succinate dehydrogenase (SDH)-deficient RCC, ELOC-mutated RCC, and anaplastic lymphoma kinase (ALK)-rearranged RCC [2].

Though a large number of all RCC diagnoses occur at early stages and may be curable with localized treatments (e.g., surgery), cancer-specific mortality drops significantly for patients who present with advanced stages that warrant systemic therapies (27–28% at 5 years) [1]. The rarity and diversity of nccRCC subtypes have made these cancers challenging to study prospectively; however, an increasing number of published and ongoing clinical trials have focused specifically on systemic therapies in these diseases. Of note, aggressive focal therapy (e.g., surgery and radiation) can play important roles in the management of nccRCC patients, and, though not discussed explicitly in this review, understanding the likelihood of response to systemic therapy can aid in personalizing treatment approaches to patients with specific disease subtypes.

The primary types of systemic therapies with prospective data in nccRCC subtypes are immune checkpoint inhibitors (ICIs), vascular endothelial growth factor receptor (VEGFR)-targeting tyrosine kinase inhibitors (TKIs), mammalian target of rapamycin (mTOR) inhibitors, vascular endothelial growth factor (VEGF)-targeting monoclonal antibodies, and chemotherapies. Mirroring the success of ICI-based doublets in ccRCC, several prospective clinical trials have tested ICI-based doublets in single-arm phase 2 trials for nccRCC patients [3]. We discuss these trials and changes to the systemic treatment landscape for nccRCC subtypes.

## 2. Results

Updates from the 2022 WHO classification of nccRCC are provided to contextualize the nccRCC systemic therapy literature published prior to these recent changes. A summary of prospective trial efficacy results for papillary, chromophobe, unclassified, and translocation RCC for TKIs and mTOR inhibitors is provided in Table 1 and for ICI monotherapies and combination strategies in Table 2. Collecting duct and renal medullary RCC data are reported separately given their unique biology and chemotherapy-based treatment strategies. Finally, other molecularly defined subtypes are discussed, with a focus on FH-deficient RCC.

The primary endpoint for most prospective trials in nccRCC subtypes is overall response rate (ORR), which reports the number of complete responses (CRs, no evidence of viable tumor) or partial responses (PRs, 30% shrinkage or more of tumor index lesions) out of the total number of patients in a trial with measurable disease. While useful as a surrogate marker of efficaciousness in early-phase trials, this outcome falls short in explaining duration of response or survival time. These metrics are better captured through reporting median progression-free survival (mPFS) or median overall survival (mOS) times, which require longer follow-up times for patients who receive a specific therapy. These metrics are provided when available for the trials discussed, and ongoing trials should continue to emphasize these meaningful outcomes in patients with nccRCC.

Additionally, patient comorbidities can play a significant role in systemic therapy selection and their sequencing for patients across subtypes of nccRCC. VEGFR-targeted TKIs and VEFR antibodies should be avoided in patients with poorly controlled hypertension, recent major cardiovascular events, or known gastrointestinal fistulas or perforations [23]. ICIs should be avoided in patients with pre-existing autoimmune conditions requiring high levels of immunosuppression, and close co-management with autoimmune disease experts is frequently required when treatment is attempted [24]. mTOR inhibitors must be used with caution in patients with poorly controlled diabetes, hyperlipidemia, or severe pulmonary disease due to risks of metabolic abnormalities and pneumonitis [25]. Local therapies such as stereotactic body radiation therapy (SBRT) can play important roles for palliation or disease control in patients with limited performance status or oligoprogressive disease [26].

### 2.1. Updates in nccRCC Classification

**Figure 1:**
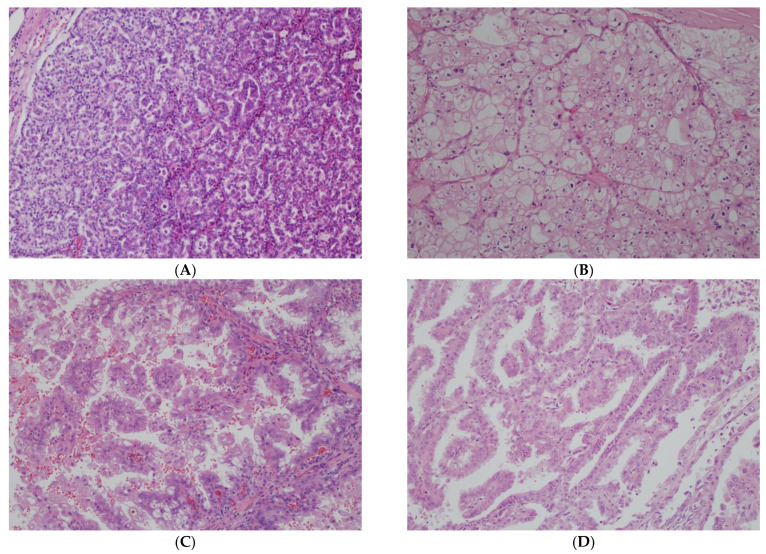
Microscopic features of morphologically defined RCCs. (**A**). Papillary RCC showing papillary architecture with fibrovascular lined by columnar cells with low-grade nuclei (typical of type 1). (**B**). Chromophobe RCC with sheets of neoplastic cells with abundant admixed clear and eosinophilic cytoplasm. The cells have prominent cell borders and wrinkled nuclei. (**C**). TFE3-rearranged RCC showing papillary architecture. Cells have abundant clear to eosinophilic cytoplasm and grade 3 nuclei. (**D**). Fumarate-hydratase-deficient RCC also demonstrating a papillary architecture. The cells have abundant eosinophilic cytoplasm and large nuclei with macronucleoli with focally perinucleolar clearing (all 100× magnification).

It is important to review recent updates in nccRCC subtype classification to contextualize the nccRCC literature that utilized prior classification schemes. The 2022 WHO RCC Classification made significant changes to how nccRCC subtypes are classified, in particular for papillary RCC [2]. A review of morphologic features of representative nccRCC subtypes is shown in Figure 1.

Prior to the 2022 WHO classification, papillary RCC was divided into type 1 and type 2 papillary RCC [27]. “Type 1” papillary RCC was defined by thin fibrovascular cores lined by a single layer of cells typically with pale cytoplasm and low-grade nuclei and high frequency of *MET* gene alterations [28]. “Type 2” papillary RCC characterized by pseudostratification, eosinophilic cytoplasm, and prominent nucleoli as well as gain in chromosomes 12, 16, and 20. Importantly, many type 2 papillary RCC show significant variability in both their morphology and clinical behavior and, with growing use of molecular studies, are now recognized as distinct molecular or histologic types, such as FH-deficient RCC, MiT family translocation RCC, ALK-rearranged RCC, and acquired cystic-disease-associated RCC (ACD-RCC) [29]. Further, recent analyses have shown that papillary RCC are better prognostically classified by the WHO grade rather than the type 1/type 2 subtyping and, therefore, in the 2022 WHO, the former “type 1” papillary RCC is regarded as the classic papillary RCC and subtyping into type 1 or 2 is no longer recommended.

Ongoing innovations in artificial intelligence may drive more accurate nccRCC classification into already existing disease subtypes as well as lead to additional methods of classification [30]. These methods will become increasingly important as subtype classification relies more on complex multimodal data ranging from histopathology to multi-omic molecular analyses.

### 2.2. Papillary RCC

Papillary RCC is believed to originate from the renal proximal tubule similar to ccRCC, though single-cell studies have highlighted that some of these tumors may originate from the collecting duct [31]. Due to the previously discussed updates in the 2022 WHO classification of papillary RCC, trial results that included this subtype must be interpreted with the context that the modern definition of papillary RCC consists largely of what was previously defined as type 1 papillary RCC, with much of type 2 papillary RCC now reclassified into new independent subtypes.

Analogous to the history of systemic therapies in advanced ccRCC, the prospective trials in papillary RCC initially focused on VEGFR TKI monotherapies, with cabozantinib (inhibitor of MET, AXL, and VEGFR2) outperforming sunitinib (inhibitor of PDGFR, VEGFR1-3, KIT, and RET) in the only direct comparison of these agents [5]. Next came ICI monotherapies and then, more recently, an increasing number of studies have evaluated ICI/ICI and VEGFR-TKI/ICI combination strategies, with single-arm prospective studies of cabozantinib with nivolumab (PD-1 antibody) and lenvatinib (inhibitor of VEGFR1-3, FGFR1-4, PDGFRA, KIT, and RET) with pembrolizumab (PD-1 antibody) showing compelling efficacies. Additional, randomized trials are ongoing and further discussed below (NCT05411081) [32].

#### 2.2.1. VEGFR TKI Monotherapy

Single-arm studies of VEGFR TKIs enrolling patients with papillary RCC are summarized in Table 1 and include the papillary-exclusive AXIPAP trial for axitinib (inhibitor of VEGFR1-3, ORR 28.6%) [4], NCT01538238 for pazopanib (inhibitor of PDGFR, VEGFR1-3, FGFR1/3, and KIT, ORR 39% for papillary subgroup) [10], NCT02915783 for lenvatinib with everolimus (mTOR inhibitor, ORR 15% for papillary subgroup) [8], and an NCT00502307 subgroup analysis for tivozanib (inhibitor of VEGFR1-3, ORR 87.5% for papillary subgroup) [11]. Though the subgroup had a small number of patients, the tivozanib study in particular showed a high response rate for papillary RCC, with seven out of the eight patients responding to treatment [11]. Additional single-arm studies include the SUPAP study for sunitinib, which divided outcomes by the historical type 1 (ORR 13%, mOS 18.7 months (mo), 95% confidence interval [CI] 5.7–26.1 mo) and type 2 (ORR 11%, mOS 12.4 mo, 95% CI 8.2–14.3 mo) papillary RCC [33].

Randomized trials of VEGFR TKI monotherapy include the ESPN and ASPEN trials comparing sunitinib to everolimus for nccRCC [6,7]. The ESPN trial enrolled 31% papillary RCC patients with an ORR of 9% for sunitinib and 2.8% for everolimus. The trial did not find a significant mPFS difference, with 6.1 mo for sunitinib and 4.1 mo for everolimus (*p* = 0.6). In the ASPEN trial, sunitinib showed a prolonged mPFS over everolimus (8.3 versus (vs.) 5.6 mo, hazard ratio (HR) 1.41, 80% confidence interval (CI) 1.03–1.92, *p* = 0.16) with an ORR of 18% vs. 9% [7]. Of the 70 patients (65%) with papillary RCC, ORR was higher at 24% for sunitinib vs. 5% with everolimus. The difference in findings between these trials may be driven by the nonpapillary subgroups, which encompassed 60% of the total patients in ESPN but only 35% in ASPEN.

More recently, the PAPMET trial randomized patients with papillary RCC to sunitinib, cabozantinib, savolitinib (MET inhibitor), and crizotinib (inhibitor of ALK, HGFR, and ROS1). Cabozantinib demonstrated an improved ORR and mPFS over sunitinib (ORR: 23% vs. 4%, two-sided *p* = 0.010; mPFS: 9.0 vs. 5.6 mo, HR 0.60, 95% CI 0.37–0.97, *p* = 0.019) [5]. The savolitinib and crizotinib arms were halted at the prespecified futility analysis. Important toxicities in the cabozantinib arm included fatigue (70%, 15% grade 3+), diarrhea (56%, 7% grade 3+), and hand–foot syndrome (49%, 21% grade 3+), with dose reductions in 34% of patients and a discontinuation rate of 23%. For sunitinib, toxicities included fatigue (62%, 9% grade 3+), diarrhea (49%, 7% grade 3+), and nausea (44%, 9%, 21% grade 3+), with dose reductions in 38% of patients and a discontinuation rate of 24%

Finally, while most studies evaluated VEGFR TKI monotherapy in the first line, the UNICAB trial was designed to address the role of cabozantinib in the post-ICI or ICI-ineligible setting. However, the trial had low recruitment in the setting of the COVID-19 pandemic. Of the total 31 patients enrolled with nccRCC (including 10 papillary type 1 and 4 papillary type 2), the ORR was 22.6% (95% CI 9.6–41.1 mo) [34].

#### 2.2.2. mTOR Inhibitors

Everolimus monotherapy has been utilized as a control arm in multiple randomized trials with relatively low efficacy in papillary RCC (Table 1). The prior single-arm phase II RAPTOR study of everolimus in papillary RCC reported similar low efficacy with an ORR of 1/88 (1%) and an mOS of 21.4 mo (95% CI 15.4–28.4 mo). In combination, the NCT02915783 phase II trial of everolimus plus lenvatinib showed a 15% ORR, while the NCT01399918 phase II trial of everolimus plus bevacizumab (VEGF antibody) showed an ORR rate of 35% in patients with treatment-naïve, papillary RCC [9].

#### 2.2.3. ICI Monotherapy

Prior to the exploration of ICI combinations, several trials investigated ICI monotherapy strategies. In the phase II KEYNOTE-427, pembrolizumab achieved an ORR of 26.7% in 165 treatment-naïve, nccRCC patients, of which 71.5% with papillary RCC achieved an ORR of 28.8% [12]. Of the responders, 59.7% had durable response lasting longer than 12 mo. The phase IIIb/IV CheckMate 374 trial evaluated nivolumab monotherapy in patients with up to three prior lines of therapy and found an ORR of 13.6% (including two partial responses (PRs) in patients with papillary RCC) and an mPFS of 2.2 mo.

The UNISoN trial was a single-arm trial evaluating nivolumab monotherapy followed by nivolumab plus ipilimumab at progression [14]. The ORR for nivolumab alone was 16.9% with 14.7% ORR across papillary RCC patients. The ORR in patients who received doublet ICI therapy after progression on nivolumab was 10%.

#### 2.2.4. ICI Combination Therapies

KEYNOTE-B61 was a multicenter single-arm phase 2 trial examining the combination of pembrolizumab and lenvatinib in advanced nccRCC [15]. In total, 93 (59%) of the 158 enrolled patients had papillary RCC, and the primary outcome of ORR for patients with papillary RCC was 54% (8 CRs and 42 PRs). mPFS was 18 months (95% CI 14 mo to Not Reached). Toxicity profiles were similar to the CLEAR trial of this ICI–TKI combination therapy in ccRCC [35], with 51% of patients having grade 3 or higher toxicities and the most common adverse events including hypertension (34% overall, 23% grade 3+), diarrhea (41% overall, 3% grade 3+), and hypothyroidism (36% overall, 1% grade 3+). Thirty-four percent of patients reduced their dose, while 14% of patients discontinued lenvatinib due to toxicity. Fifteen percent of patients discontinued pembrolizumab and seven percent discontinued both drugs due to toxicity.

CA209-9KU was a single-center phase II trial evaluating the combination of nivolumab and cabozantinib in advanced nccRCC [16]. A total of 32 of the 47 enrolled patients had papillary RCC with an ORR of 47% (95% CI 30–64%, 1 CR, and 14 PR) and an mPFS of 13 mo (95% CI 7–16 mo) [36]. Across all enrolled patients, toxicity profiles were similar to the CheckMate 9ER trial of this IO–TKI combination in ccRCC [37], with 55% grade 3 or above toxicities. The most common adverse events were fatigue (70%, 0% grade 3+), palmar–plantar erythrodysesthesia (68%, 5% grade 3+), and diarrhea (63%, 7.5% grade 3+). Of the patients, 88% required a dose reduction, 40% discontinued cabozantinib, 40% discontinued nivolumab, and 33% discontinued both drugs due to side effects.

Prospective trials of ICI–ICI combination therapy in nccRCC include the multicohort CheckMate 920 phase 3b/4 study of nivolumab plus ipilimumab (anti-CTLA4 antibody). This trial enrolled patients excluded from registrational Checkmate studies and included an nccRCC cohort [17]. Detailed subtype-level outcomes are not available, though, of the 52 patients enrolled, 18 had papillary RCC. In the overall, response-evaluable cohort of 46 patients, ORR was 19.6% (95% CI 9.4–33.9), mPFS was 3.7 mo (95% CI 2.7–4.6 mo), and mOS was 21.2 mo (95% CI 16.6 mo—not evaluable). Within the papillary RCC cohort, one patient achieved a CR and four patients achieved PRs. Overall toxicities were consistent with the CheckMate 214 trial of this ICI–ICI combination in ccRCC [38], with 36.5% of patients having grade 3 or higher toxicities. The most common toxicities were fatigue (48.1%, 3.8% grade 3+), diarrhea (30.8%, 3.8% grade 3+), and nausea (26.9%, 0% grade 3+).

The SUNNIFORECAST trial offered additional prospective data for the nivolumab/ipilimumab combination [18]. This multicenter phase II trial randomized 309 patients with nccRCC, including 178 patients (57.6%) with papillary RCC, to either nivolumab plus ipilimumab or standard of care (SOC). In the SOC arm of 152 patients, 124 received TKI monotherapy and 17 received ICI–TKI combination therapy. The primary outcome was 12-month overall survival (OS) rate, for which the nivolumab plus ipilimumab arm outperformed the SOC arm (86.9% vs. 76.8%, *p* = 0.014). However, the OS rates at 6 mo (94.7% vs. 90.0%, *p* = 0.067), the OS rates at 18 mo (76.6% vs. 69.1%, *p* = 0.084), and the mOS of 42.4 vs. 33.9 mo did not reach statistical significance (*p* = 0.292). The ORR was 32.8% in the nivolumab/ipilimumab group vs. 19.6% in the SOC group and 29.2% vs. 21.0% in the papillary RCC subgroup. In the subgroup analysis, for the 26 patients whose tumor had a PD-L1 expression of greater than or equal to 1%, there was a trend towards improved OS, HR 0.56 (95% CI 0.33–0.95).

Outside of ICI–ICI and ICI–TKI combinations, additional ICI combinations have been explored in papillary RCC. These include savolitinib with durvalumab (PD-L1 antibody) in the CALYPSO trial [20], cabozantinib with atezolizumab (PD-L1 antibody) in COSMIC-021 [21], and bevacizumab with atezolizumab in NCT02724878 [22]. Notably, the CALYPSO trial of savolitinib with durvalumab had an overall ORR of 29% with a higher ORR of 53% in the MET-driven subgroup, defined as chromosome 7 gain, MET amplification, MET kinase domain variations, or hepatocyte growth factor amplification. The ongoing phase III SAMETA trial compares savolitinib plus durvalumab with control arms of sunitinib or durvalumab monotherapy in MET-driven papillary RCC [39].

The CAN-I trial (NCT04413123) investigating the triplet cabozantinib, nivolumab, and ipilimumab combination in nccRCC reported data from the first 39 treated patients, with an ORR of 21% and an mPFS of 9.7 mo (95% CI 6.1–12.7) [40]. The Alliance ICONIC trial investigates the same triplet therapy in rare genitourinary cancers, including nccRCC subsets of collecting duct RCC, sarcomatoid RCC, papillary RCC, chromophobe RCC, and renal medullary cancer [41]. The SWOG S2200/PAPMET2 (NCT05411081), randomized trial of cabozantinib with or without atezolizumab for patients with papillary RCC may provide additional insights [42].

#### 2.2.5. Papillary RCC Summary

Though existing trials in papillary RCC are largely single-arm and rely on outdated classification criteria to define papillary RCC, they demonstrate compelling efficacy for the TKI–ICI combinations, including cabozantinib/nivolumab and lenvatinib/pembrolizumab. Additionally, randomized data from the PAPMET trial support the use of cabozantinib monotherapy for this subtype, and the ongoing, randomized, PAPMET2 investigates cabozantinib with or without atezolizumab. Increasing evidence, including the recent SUNNIFORECAST study, highlights ICI–ICI combination therapy as another option in this disease subtype. Efficacy interpretation of ICI-based approaches, especially ICI monotherapy or ICI–ICI combination therapy, is limited by reliance on metrics such as ORR, when it may be better assessed by outcomes such as response duration and overall survival. Future directions for this nccRCC subtype include understanding how to stratify treatment by biomarkers such as MET-driven disease, as well as sequence lines of therapies, given the majority of these studies occurred in the treatment-naïve setting. Ongoing trials in papillary RCC and other nccRCC subtypes are summarized in Table 3.

### 2.3. Chromophobe RCC

Chromophobe RCC makes up 5% of RCC cases and arises from the collecting duct intercalated cells [47]. It is associated with chromosomal aneuploidy, as well as *TP53* and *PTEN* mutations. In addition, the folliculin gene (*FLCN*) is mutated in the context of Birt–Hogg–Dube syndrome and the *TSC1* or *TSC2* genes are mutated in the context of tuberous sclerosis complex [48]. While the majority of chromophobe RCC tumors do not metastasize, they can dedifferentiate into more aggressive subtypes; this process is typically associated with *TP53* mutation and whole-genome duplication [49].

No prospective clinical trials have focused solely on chromophobe RCC and, given the relative rarity of this disease, collective nccRCC trials do not selectively test treatments for chromophobe RCC. Of the nccRCC trials with a chromophobe cohort (Table 1), efficacy varies widely, with some demonstrating promising efficacy, while others show no response. Compared to papillary and other nccRCC subtypes, chromophobe RCC patients seem to have higher responses to mTOR inhibition. For example, everolimus monotherapy showed a 33% ORR (2/6 patients) in the ASPEN trial, while lenvatinib and everolimus showed a 44.4% ORR (4/9 patients) in NCT02915783 [7,8].

Response to ICI monotherapy and ICI combination therapy varies widely. With ICI monotherapy, a 9.5% ORR (2/21 patients) to pembrolizumab in KEYNOTE-427, a 28.6% ORR (2/7 patients) to nivolumab in Checkmate 374, and a 7.7% ORR (1/13 patients) to nivolumab in UNISoN was observed [12,50,51]. With ICI–TKI and ICI–ICI combination therapies, no response was seen in CA209-9KU with cabozantinib–nivolumab and only one response (ORR 9.1%) was seen in NCT04413123 with cabozantinib–nivolumab–ipilimumab [16,40]. Conversely, a 28% ORR (8/29 patients) was seen in KEYNOTE-B61 with lenvatinib and pembrolizumab and a 25.9% ORR (7/27 patients) was seen in SUNNIFORECAST with nivolumab–ipilimumab [15,18]. Additional meaningful ICI efficacy endpoints such as duration of response and OS are not yet available.

Several retrospective reviews supplement the paucity of data in prospective trials for this disease and report similar ORR rates to subgroups of prospective trials (see Table 4). A retrospective review by Moussa et al. showed activity for nivolumab–ipilimumab in chromophobe RCC (ORR 25% in 3/12 patients), and a review specific to chromophobe RCC demonstrated responses to this disease with TKI monotherapy (ORR 15%), ICI–TKI combination therapy (30%), and ICI–ICI combination therapy (ORR 12.5%) [52,53]. Two retrospective reviews on cabozantinib use in nccRCC demonstrated ORR rates for chromophobe RCC patients of 30% (3/10 patients) and 17% (1/6 patients) [54,55].

The biology of chromophobe RCC is vastly different from papillary RCC and other nccRCC subtypes and, although some responses have been shown with existing treatment strategies, generally, prognosis is poor for metastatic disease. Recent work on the drivers of tumorigenesis in chromophobe RCC has highlighted the role of both IL-15 and ferroptosis in the disease, and future treatments targeting these pathways could be considered [48].

### 2.4. Not Otherwise Specified (Also Referred to in the Literature as Unclassified) RCC

RCC-NOS is a diverse group of RCC that do not fit into currently recognized diagnostic categories of RCC. These tumors generally have a heterogeneous morphology/immunohistochemistry (IHC) profile and typically include high-grade RCCs. Pure sarcomatoid morphology, where a differentiated component has been completely effaced, may also be included [58]. These tumors often need liberal sampling and IHC workup to rule out extrarenal and urothelial-origin tumors. With increasing use of molecular studies, this category has become infrequent. In the 2022 WHO classification, unclassified RCC has been renamed to RCC-NOS to emphasize that it is not a distinct subtype [2].

Similar to chromophobe RCC, no prospective clinical trials have included solely RCC-NOS, and retrospective studies mirror the ORRs reported in subgroups of prospective trials [52,54]. Treatment recommendations for this subtype largely mirror that of papillary RCC, with options including ICI–TKI combination strategies or cabozantinib monotherapy. Additionally, the heterogeneity of this subtype may mask ccRCC biology, with one cohort reporting 11.9% of unclassified RCC patients harboring a VHL mutation [59]. Thus, systemic treatment approaches used in ccRCC may be considered, including ICI–ICI combination therapy.

### 2.5. Translocation RCC

Translocation RCC, or microphthalmia-associated transcription factor family translocation RCC (MiTF-tRCC), encompasses a rare group of diseases involving translocations affecting the short arm of the X chromosomes and fusions of the TFE3, TFEB, or MITF genes [60,61]. In the 2022 WHO classification, this disease subtype has been broken up further into molecularly defined entities including TFE3-rearranged RCC (Xp11 translocation RCC) and TFEB-altered RCC, given the different behaviors of these specific subtypes [2]. TFEB-altered RCC includes TFEB-rearranged RCC and TFEB-amplified RCC. TFEB-amplification RCC was recently described harboring amplification of the 6p21 region with resultant TFEB overexpression [62]. TFEB-amplified RCC occurs in older patients and has a worse outcome. Morphologically, translocation RCC can often mimic other common RCCs such as clear cell and papillary RCC and are, therefore, often misdiagnosed. For example, in IMmotion151, more than 15 tumors classified as ccRCC were found to harbor *TFE* fusions [63]. Molecular subset clustering from this trial suggests these tumors may have a permissive immune microenvironment; however, significant benefit from immune checkpoint inhibitor-based regimens has not been consistently observed.

As with chromophobe and unclassified RCCs, assessment of response in translocation RCCs is limited to subgroup analysis of nccRCC trials. ICI–TKI combination approaches appear promising, with 4/6 translocation RCC patients responding to lenvatinib plus pembrolizumab in KEYNOTE-B61 and 1/2 translocation RCC patients responding to cabozantinib plus nivolumab in CA209-9KU [15,36], but the small numbers of patients with this subtype limit the generalizability of these findings. Two retrospective reviews of 29 and 22 translocation RCC patients demonstrated ORRs of 5.5% and 14% for ICI–ICI combination therapy and ORRs of 36% and 54% for ICI–TKI combination therapies [56,57]. The phase II NCT03595124 trial is prospectively investigating the activity of nivolumab with or without axitinib specifically in patients with TFE/translocation RCC and is estimated to complete accrual in 2026 [43]. Ongoing investigations into how the fusions that define this nccRCC subtype drive tumorigenesis have highlighted MET and RET kinases as potential pathways for therapeutic targets [64].

### 2.6. Collecting Duct Carcinoma

Collecting duct carcinoma arises from the distal convoluted tubules and generally presents at advanced or metastatic stages. These tumors can involve mutations in *NF2*, *SETD2*, *SMARCB1*, and *CDKN2A* [65,66]. Histologically, collecting duct carcinoma is a diagnosis that requires diligent exclusion from another epithelial tumor, urothelial carcinoma, SMARCB1-deficient renal medullary carcinoma (RMC), FH-deficient RCC, and high-grade RCC and is vanishingly rare. Treatments for collecting duct carcinoma traditionally involve chemotherapy, based on biologic similarity to urothelial cancers, with systemic therapy trials summarized in Table 5.

Prospective data for modern chemotherapies include the phase II GETUG study evaluating gemcitabine plus a platinum chemotherapy agent, which found an ORR of 6/23 (26%, 95% CI 8–44%) [67]. The addition of sorafenib (inhibitor of VEGFR1-3, PDGFR, and RAF kinases) to chemotherapy was studied in a single-arm phase 2 study that showed an mPFS of 8.8 mo (95% CI 6.7–10.9 mo) and ORR of 30.8% in 26 patients with collecting duct carcinoma. The phase II BEVABEL-GETUG/AFU24 trial examined the addition of bevacizumab to gemcitabine and platinum chemotherapy and found an ORR of 41.2% (95% CI 25–57%), with an mPFS of 5.9 mo (5.3–9.3 mo) and mOS of 11.1 mo (7.6–24.2 mo) [68].

More recently, the BONSAI trial showed the efficacy of first-line cabozantinib monotherapy, which achieved an ORR of 35% (95% CI 16–57%) and an mPFS of 4 months (95% CI 3–13 mo) in 23 patients with collecting duct carcinoma [69]. Collecting duct carcinoma was excluded from all prospective nccRCC IO–TKI combination trials, and only two patients in the IO–IO CheckMate 920 trial had collecting duct RCC, with one patient having a PR [17]. Nine patients in the SUNNIFORECAST trial evaluating ICI–ICI combination therapy had collecting duct carcinoma, although a subgroup analysis is not yet available [18].

**Table 5:** Prospective and retrospective data in collecting duct and renal medullary carcinoma.

Study	Study Drug(s)	n	nccRCC Subtype (s)	ORR (95% CI)	mPFS (95% CI)
Prospective Clinical Trials					
BEVABEL-GETUG/ AFU24 [68]	Gemcitabine + platinum chemo + bevacizumab	34	Collecting Duct Renal Medullary	41.2% (25–57%)	5.9 mo (5.3–9.3 mo)
GETUG 2007 [67]	Gemcitabine + platinum chemo	23	Collecting Duct	26% (8–44%)	7.1 mo (3–11.3 mo)
BONSAI [69]	Cabozantinib	22	Collecting Duct	35% (16–57%)	4 mo (3–31 mo)
BONSAI-2 [70]	Nivolumab (Post-cabo)	4		25%	PFS 2.8–19.9 mo
NCT02721732 [71]	Pembrolizumab (1 front-line)	5	Renal Medullary	0/5 (0%)	8.7 weeks
Retrospective Reviews					
Shah et al. [72] (at any line of therapy)	Carboplatin/ paclitaxel (±bev)	21	Renal Medullary	7/21 33%	N/A
	Gemcitabine/ cisplatin (±bev)	12		4/12 33%	N/A
	Gemcitabine/ doxorubicin (±paclitaxel/bev)	11		3/11 27.3%	N/A
	Gemcitabine/ carboplatin/ paclitaxel	2		1/2 50%	N/A
	Gemcitabine/ cisplatin/ paclitaxel	1		1/1 100%	N/A
Wilson et al. [73]	Gemcitabine + doxorubicin (second-line)	16	Renal Medullary	18.8%	2.8 mo (0–6 mo)

Bev—bevacizumab, CI—confidence interval, ICI—immune checkpoint inhibitor, mo—month, TKI—tyrosine kinase inhibitor, N/A—not available, NR—not reached, ORR—overall response rate.

### 2.7. Renal Medullary Carcinoma

RMC is an aggressive malignancy arising from the collecting duct and is associated with sickle hemoglobinopathies. In the latest 2022 WHO classification system, RMC is now a molecularly defined nccRCC subtype characterized by loss of *SMARCB1* expression [74]. Patients with RMC have been largely excluded from nccRCC trials, and RMCs generally have poor response to treatments effective for other RCCs such as VEGFR TKIs [72,75]. Trial and review data for RMC are summarized in Table 5. Based on available data, platinum-based chemotherapy with or without bevacizumab is the mainstay first-line systemic therapy, though responses are generally not durable and outcomes are poor across all stages of disease [68,72].

A phase II basket trial evaluating pembrolizumab in rare cancers included five patients with RMC, none of whom had a significant response [71]. Ongoing trials are investigating ICI-based combination therapies, including NCT05347212 with nivolumab plus relatlimab (LAG3 antibody) in RMC and NCT05286801 with tiragolumab (TIGIT antibody) plus atezolizumab in *SMARCB1-* or *SMARCA4*-deficient RCC [44,45].

### 2.8. Fumarate-Hydratase-Deficient RCC and Other Molecularly Defined nccRCC Subtypes

A molecularly defined subtype of nccRCC is FH-deficient RCC, which is driven by a sporadic or hereditary *FH* mutation [76]. A hereditary form of the disease can be associated with hereditary leiomyomatosis and renal cell cancer (HLRCC) and includes a subgroup of what was previously defined as type 2 papillary RCCs.

A phase II study of bevacizumab and erlotinib (epidermal growth factor receptor (EGFR) inhibitor) that enriched for patients with FH-deficient RCCs (either HLRCC or papillary RCC) reported an overall ORR of 51% (42/83 patients, CI 40–61%) and an HLRCC cohort ORR of 64% (27/42 patients, 95% CI 49–77%) [77]. Biochemically, FH-deficient tumors have accumulation of hypoxia-inducible factor (HIF) that can upregulate VEGF and be stimulated by EGFR pathways, leading to trials studying both EGFR and VEGF blockade for patients with these tumors [76]. Ongoing trials exploring expansion upon this dual blockade strategy include NCT04981509 examining bevacizumab, erlotinib, and atezolizumab for patients with HLRCC or papillary RCC [46].

Regarding ICI combination therapies, a phase II trial of the poly ADP ribose polymerase inhibitor (PARPi) talazoparib combined with avelumab (PD-L1 antibody) in genomically defined metastatic kidney cancer reported an ORR of 0% in a cohort of eight patients that included four FH-deficient RCC and one SDH-deficient RCC [78]. Of note, six had previously received ICI in prior lines of therapy. Recently, encouraging phase II data were presented on the ICI–TKI combination of lenvatinib plus tislelizumab (PD-1 antibody) in FH-deficient RCC tumors, which showed an ORR of 93.3% (14/15 patients), including CRs in 3 of 15 patients (20%) [79]. These approaches highlight the importance of ongoing studies for ICI combination strategies in this molecular subtype.

Emphasizing the value of thorough molecular characterization and consideration of additional molecular-targeting approaches for rare subtypes of nccRCC, Pal et al. highlight three case reports of ALK-rearranged RCC having excellent responses to the ALK kinase inhibitor alectinib, even after multiple other lines of other systemic therapies [80]. Thorough molecular characterization of tissue samples and/or tumor cell free DNA (cfDNA) provides additional opportunities to identify targeted approaches to treating patients with these rare cancers, especially with an increasing number of tumor-agnostic predictive molecular biomarkers for specific systemic therapies [81].

### 2.9. Sarcomatoid Differentiation and PD-L1 Status

The presence of sarcomatoid differentiation may be a useful predictive biomarker of ICI response in patients with ccRCC [82]. Several ICI monotherapy and ICI combination studies in nccRCC also provide subgroup outcomes for patients with sarcomatoid differentiation and PD-L1 expression ≥ 1% (Table 6). Just as with ccRCC, sarcomatoid differentiation appears to be a useful biomarker for increased responsiveness to ICI-based therapies. However, no generalizable statements can be taken from these single-arm studies with multiple layers of heterogeneity (e.g., varying histologic subtypes and simultaneous TKI treatments). PD-L1 positivity (PD-L1 ≥ 1%) may also predict improved immunotherapy responses in nccRCC subtypes and, of particular interest, was associated with improved OS for patients treated with nivolumab–ipilimumab in the SUNNIFORECAST trial (HR 0.56, 95% CI 0.33–0.95). Future studies in nccRCC subtypes should continue to investigate these biomarkers when including ICI-based interventions.

## 3. Discussion

Non-clear cell RCC encompasses a diverse set of malignancies with varying response to different classes and combinations of systemic therapies. The diversity of these diseases may not be adequately reflected in clinical guidelines. For example, the current NCCN guidelines do not differentiate among nccRCC subtypes for preferred systemic therapy regimens, which include clinical trial, cabozantinib, cabozantinib with nivolumab, or lenvatinib with pembrolizumab [83]. Evidence for these recommendations mainly comes from single-arm studies where the largest enrolling subtype is papillary RCC. The rarity of most nccRCC subtypes make clinical trial design in these diseases challenging, particularly in the locally advanced or metastatic setting, where systemic therapies are frequently warranted. However, more precise nccRCC classification, especially molecularly defined disease subtypes, opens the doors to an increasing number of collaborative multicenter and innovative trial designs that have shown benefit across a variety of rare cancers [84]. The nuances of nccRCC subtypes discussed across this review, in particular the unique biology of subtypes such as collecting duct carcinoma and renal medullary carcinoma, argue against systemic treatment guidelines aimed to encompass all nccRCC subtypes.

Acknowledging the limitations of available evidence, TKI–ICI combinations (pembrolizumab with lenvatinib or nivolumab with cabozantinib) or cabozantinib monotherapy present the most compelling evidence for first-line treatment in papillary RCC. The randomized PAPMET trial of papillary RCC patients demonstrated a benefit of cabozantinib over sunitinib, and the ongoing randomized PAPMET2 trial is exploring the benefit of cabozantinib/atezolizumab when compared to cabozantinib monotherapy. Systemic treatment options for chromophobe RCC, unclassified RCC, and translocation RCC are largely similar to papillary RCC, though evidence is limited to subgroup analyses of nccRCC trials and retrospective reviews. While everolimus-based treatments have shown encouraging activity in patients with chromophobe RCC, additional insights into the unique biology of this disease may present opportunities to expand treatment options. Similarly, for translocation RCC, ongoing investigations into the molecular fusions that drive this disease may open up novel treatment targets. The ICI–ICI combination of nivolumab with ipilimumab has growing evidence for use across specific nccRCC subtypes (papillary, chromophobe, unclassified, and translocation RCC), and the potential for durable responses may not be adequately reflected in commonly reported efficacy outcomes such as ORR. Long-term follow-up for meaningful ICI efficacy endpoints, such as duration of response and overall survival, may shed additional light.

Available evidence for managing collecting duct RCC and RMC is less robust and comes from small prospective trials and retrospective reports. Based on these data, an effective treatment in both subtypes includes platinum-based chemotherapy combinations with consideration for the addition of bevacizumab. In collecting duct RCC, there are prospective data to consider cabozantinib monotherapy as an alternative treatment option. Additional subgroup analyses of ongoing and future studies will demonstrate whether ICI combination strategies are effective in these diseases. For RMC patients, classes of systemic treatments outside of chemotherapy such as ICIs and TKIs have not yet demonstrated effectiveness, though ongoing trials are investigating novel combinations of these therapies.

Finally, the 2022 WHO classification of RCC added additional molecular-based subtypes that may expand precision oncology approaches for nccRCC systemic therapies. Joint EGFR and VEFR inhibition with bevacizumab and erlotinib in FH-deficient RCC has proven a promising approach, and ICI combinations appear effective in this disease subtype as well. Tumor-agnostic predictive biomarkers may provide additional treatment options for nccRCC patients, highlighting the importance of thorough molecular testing for these tumors. Ongoing studies in nccRCC have begun to align with molecular characteristics of specific nccRCC subtypes, such as MET-driven papillary RCC.

## 4. Conclusions

Currently available clinical trial data highlight ICI–TKI combination treatment (lenvatinib plus pembrolizumab or cabozantinib plus nivolumab) or cabozantinib monotherapy as the systemic therapy options with the strongest evidence for papillary RCC, unclassified RCC, chromophobe, and translocation RCC. A growing body of literature highlights that ICI–ICI combination therapies may be considered in these nccRCC subtypes. Additionally, chromophobe RCC may benefit from everolimus-based approaches. For both chromophobe RCC and translocation RCC, translational discoveries into their unique biologies are opening up new targets for future treatments. Platinum-based chemotherapy combinations remain the best available systemic therapies for collecting duct RCC and RMC; cabozantinib monotherapy may be an additional option for collecting duct RCC. Thorough molecular characterization of nccRCC tumors is essential for evaluating potential precision oncology treatment approaches.

## Figures and Tables

**Table 1:** Prospective clinical trials for TKIs and mTOR inhibitors in papillary, chromophobe, unclassified, and translocation RCCs.

Trial Name	Study Drug(s)	n	Efficacy (ORR *, mPFS, mOS with 95% CI)			
Papillary RCC-Only Trials						
AXIPAP [4]	Axitinib	44	ORR 12/42, 28.6% (15.7–44.6) mPFS 6.6 mo (5.5–9.2)			
PAPMET [5]	Cabozantinib	44	ORR 23% mPFS 9.0 mo (6–12) mOS 21.5 mo (12.0–28.1)			
	Sunitinib	46	ORR 4% mPFS 5.6 mo (3) mOS 17.3 mo (12.8–21.8)			
	Savolitinib	29	ORR 3%			
	Crizotinib	28	ORR 0%			
Trials With Multiple nccRCC Subtypes						
			Papillary RCC	Chromophobe RCC	Unclassified RCC	Translocation RCC
ESPN [6]	Sunitinib	34	ORR 3/33 (9%)			
			mPFS 5.7 mo (1.4–19.8)	mPFS 8.9 mo (2.9–20.1)	mPFS 9.4 mo (3.3–15.4)	mPFS 6.1 mo (6.0–8.8)
	Everolimus	38	ORR 1/35 (3%)			
			mPFS 4.1 mo (1.5–7.48)	NA	mPFS 4.7 mo (2.6–NA)	mPFS 3.0 (1.3–NA)
ASPEN [7]	Sunitinib	51	ORR 8/33 24%	ORR 1/10 10%	ORR 0/8 0%	-
	Everolimus	57	ORR 2/37 5%	ORR 2/6 33%	ORR 1/14 7%	-
NCT02915783 [8]	Lenvatinib + everolimus	31	ORR 3/20 15%	ORR 4/9 44.4%	ORR 1/2 50%	-
NCT01399918 [9]	Everolimus + Bevacizumab	35	ORR 7/18 ** 38.9%	ORR 2/5 40%	ORR 1/9 11.1%	-
NCT01538238 [10]	Pazopanib	28	ORR 7/18 39%	ORR 1/3 33%	ORR 0/2 0%	NA
NCT00502307 [11]	Tivozanib	46	ORR 7/8 87.5%	ORR 2/2 100%	ORR 18/27 66.7%	-

* ORR can only be estimated for a subsection of the total n with measurable disease. ** Includes unspecified RCC with papillary RCC features. CI—confidence intervals, Mo—month, mOS—median overall survival, mPFS—median progression-free survival, NA—not available, ORR—overall response rate, RCC—renal cell carcinoma.

**Table 2:** Prospective clinical trials for immune checkpoint inhibitor monotherapies and combination therapies in papillary, chromophobe, unclassified, and translocation RCCs.

Trial Name	Study Drug(s)	n	Efficacy (ORR *, mPFS with 95% CI)			
			Papillary RCC	Chromophobe RCC	Unclassified RCC	Translocation RCC
Immune Checkpoint Inhibitor Monotherapy						
KEYNOTE-427 [12]	Pembrolizumab	165	ORR 34/118 28.8% (20.8–37.9)	ORR 2/21 9.5% (1.2–30.4)	ORR 8/26 30.8% (14.3–51.8)	NA
CheckMate 374 [13]	Nivolumab	44	ORR 2/24 8.3%	ORR 2/7 28.6%	ORR 1/8 12.5%	NA
UNISoN [14]	Nivolumab	80	ORR 5/34 14.7%	ORR 1/13 8%	ORR 1/8 13%	ORR 2/5 40%
	Nivolumab + Ipilimumab (post-nivo)	41	ORR 10% (95% CI 3–23)			
Immune Checkpoint Inhibitor Combination Therapies						
KEYNOTE-B61 [15]	Lenvatinib + Pembrolizumab	158	ORR 50/93 54% (43–64) mPFS 17.5 mo (15-NR)	ORR 8/29 28% (13–64) mPFS 12.5 mo (3.9-NR)	ORR 11/21 52% (30–74)	ORR 4/6 67% (22–96)
CA209–9KU [16]	Cabozantinib + Nivolumab	47	ORR 15/32 47% (30–64) mPFS 13 mo (7–16)	ORR 0/7 0% (0–41.0)	ORR 3/6 50% (12–88) mPFS 8 mo (1-NE)	ORR 1/2 50% (1–99) mPFS 14 mo (5–23)
CheckMate 920 [17]	Nivolumab + Ipilimumab	46	Overall ORR 9/46 19.6% (9.4–33.9%)			
			1 CR, 4 PR 18 patients **	0 CR, 0 PR 7 patients **	1 CR, 3 PR 22 patients **	0 CR, 0 PR 2 patients **
SUNNIFORECAST [18] (intervention arm)	Nivolumab + Ipilimumab	125	ORR 21/72 29.2%	ORR 7/27 25.9%	NA	NA
NCT04413123 [19]	Cabozantinib + nivolumab + ipilimumab	40	ORR 6/19 31.6%	ORR 1/11 9.1%	ORR 1/1 100%	ORR 0/5 0%
CALYPSO [20] MET-driven subgroup	Savolitinib + Durvalumab	41	ORR 12/41 29% (16–46)	-	-	-
		17	ORR 9/17 53% (28–77)	-	-	-
COSMIC-021 [21]	Cabozantinib + Atezolizumab	32	ORR 7/15 47%	ORR 1/9 11%	-	-
NCT02724878 [22]	Bevacizumab + Atezolizumab	42	ORR 3/12 25%	ORR 1/10 10%	ORR 3/9 3.3%	ORR 1/5 20%

* ORR can only be estimated for a subsection of the total n with measurable disease. ** Breakdown of patients with measurable disease by subtype not provided; unable to calculate ORR. CI—confidence interval, CR—complete response, PR—partial response, mo—month, mPFS—median progression-free survival, NA—not available, NE—not estimable, nivo—nivolumab, NR—not reached, ORR—overall response rate, RCC—renal cell carcinoma.

**Table 3:** Select ongoing clinical trials for subtypes of nccRCC.

Study	Study Drug(s)	nccRCC Subtype(s)	Primary Outcome	Goal Recruitment (n)	Estimated Completion
ICONIC [41] (NCT03866382)	Nivolumab, Ipilimumab, and cabozantinib	Papillary Chromophobe Collecting Duct Sarcomatoid (~50%) Renal Medullary	ORR Secondary: PFS, OS	314	February 2026
PAPMET2 [42] (NCT05411081)	Cabozantinib +/- Atezolizumab	Papillary	PFS Secondary: OS, ORR	200	July 2027
SAMETA [39] (NCT05043090)	Durvalumab +/- savolitinib vs. sunitinib	MET-driven Papillary	mPFS Secondary: OS, ORR	147	June 2026
NCT03595124 [43]	Axitinib +/- Nivolumab	Translocation	mPFS Secondary: toxicities	15	January 2026
NCT05347212 [44]	Nivolumab + Relatlimab	Renal Medullary	ORR Secondary: OS, PFS	30	July 2027
NCT05286801 [45]	Tiragolumab + atezolizumab	Renal Medullary	ORR Secondary: PFS, OS, PK	86	June 2026
NCT04981509 [46]	Bevacizumab + erlotinib + atezolizumab	Papillary HLRCC	CR Secondary: ORR,	65	December 2027

CR—complete response, HLRCC—hereditary leiomyomatosis and renal cell carcinoma, ORR—overall response rate, OS—overall survival, PK—pharmacokinetics, PFS—progression-free survival.

**Table 4:** Select retrospective reviews for subtypes of nccRCC.

Study	Study Drug(s)	n	nccRCC Subtype(s)	ORR	mPFS (95% CI)
Moussa et al. [52]	Nivolumab + Ipilimumab	55	Papillary Chromophobe Unclassified	12/25 (48%) 3/12 (25%) 5/18 (27.8%)	10.6 mo (2.8–22.8) 3.6 mo (0.9–NE) 3 mo (2.1–7)
Alhalabi et al. [56]	ICI + TKI	18	Translocation	1/18 (5.5%)	5.4 mo
	ICI + ICI	11		4/11(36%)	2.8 mo
Ged et al. [57]	ICI + TKI ICI + ICI	14 8	Translocation	1/7 (14%) 6/11 (54%)	TTF 1.2 mo TTF 6.2 mo
Paffenholz et al. [53]	ICI + TKI	10	Chromophobe	3/10 (30%)	8 mo (0–19.2)
	ICI + ICI	8		1/8 (12.5%)	3 mo (2.4–3.6)
	TKI	13		2/13 (15%)	4 mo (0–10.2)
Chanza et al. [54]	Cabozantinib	112	Papillary Chromophobe Unclassified Translocation	18/66 (27%) 3/10 (30%) 2/15 (13%) 5/17 (29%)	6.9 mo (4.6–10.1) 5.7 mo (1.1–7.8) 6.0 mo (1.4–9.9) 8.3 mo (4.6–NR)
Campbell et al. [55]	Cabozantinib	30	Papillary Chromophobe Unclassified Translocation	1/15 (7%) 1/6 (17%) 1/3 (33%) 0/2 (0%)	8.6 mo (6.1–14.7)

CI—confidence interval, ICI—immune checkpoint inhibitor, mo—month, NE—not estimable, NR—not reached, ORR—overall response rate, TKI—tyrosine kinase inhibitor, TTF—time to treatment failure.

**Table 6:** ORR by sarcomatoid differentiation and PD-L1 status in nccRCC ICI trials.

Trial Name	Study Drug(s)	n	Objective Response Rates (ORR) *		
			Overall	Sarcomatoid Differentiation	PD-L1 ≥ 1%
Immune Checkpoint Inhibitor Monotherapy					
KEYNOTE-427 [12]	Pembrolizumab	165	26.7%	16/38 42.1% (95% CI, 26.3–59.2%)	36/102 35.3% (26.1–45.4)
CheckMate 374 [13]	Nivolumab	44	13.6%	2/4 50%	NA mOS 16.3 mo
UNISoN [14]	Nivolumab	80	16.9%	NA	NA
Immune Checkpoint Inhibitor Combination Therapies					
KEYNOTE-B61 [15]	Lenvatinib + Pembrolizumab	158	49%	9/18 47% (95% CI 24–71)	54/93 58% (95% CI 47–68)
CA209-9KU [16]	Cabozantinib + Nivolumab	47	47.5%	NA	NA
CheckMate 920 [17]	Nivolumab + Ipilimumab	46	19.6%	5/14 35.7% (95% CI 12.8–64.9)	4/13 30.8%
SUNNIFORECAST [18]	Nivolumab + Ipilimumab	125	32.8%	NA	NA HR for OS 0.56 (95% CI 0.33–0.95)
NCT04413123 [19]	Cabozantinib + nivolumab + ipilimumab	40	21%	3/9 33.3%	NA
CALYPSO [20]	Savolitinib + Durvalumab	41	29%	NA	9/27 33% (95% CI 17–54)
COSMIC-021 [21]	Cabozantinib + Atezolizumab	32	23%	NA	5/23 22%
NCT02724878 [22]	Bevacizumab + Atezolizumab	42	26%	3/8 38%	9/23 36%

* ORR can only be estimated for a subsection of the total n with measurable disease. Mo—month, NA—not available, nivo—nivolumab, ORR—overall response rate, OS—overall survival, RCC—renal cell carcinoma.

## Data Availability

Not applicable.

## Abbreviations

The following abbreviations are used in this manuscript:

ALK	Anaplastic lymphoma kinase
ccRCC	Clear cell renal cell carcinoma
CI	Confidence interval
EGFR	Epidermal growth factor receptor
FH	Fumarate hydratase
HR	Hazard ratio
ICI	Immune checkpoint inhibitor
IHC	Immunohistochemistry
mOS	Median overall survival
mPFS	Median progression-free survival
mTOR	Mammalian target of rapamycin
nccRCC	Non-clear cell renal cell carcinoma
ORR	Overall response rate
OS	Overall survival
PARPi	Poly ADP ribose polymerase inhibitor
RCC	Renal cell carcinoma
SDH	Succinate Dehydrogenase
SOC	Standard of Care
TKI	Tyrosine kinase inhibitor
VEGFR	Vascular endothelial growth factor receptor
WHO	World Health Organization
